# Effectiveness of Psychological Support to Healthcare Workers by the Occupational Health Service: A Pilot Experience

**DOI:** 10.3390/healthcare9060732

**Published:** 2021-06-14

**Authors:** Guendalina Dalmasso, Reparata Rosa Di Prinzio, Francesco Gilardi, Federica De Falco, Maria Rosaria Vinci, Vincenzo Camisa, Annapaola Santoro, Daniela Casasanta, Massimiliano Raponi, Gabriele Giorgi, Nicola Magnavita, Salvatore Zaffina

**Affiliations:** 1 Health Directorate, Bambino Gesù Children’s Hospital IRCCS, 00146 Roma, Italy; guendalina.dalmasso@opbg.net (G.D.); massimiliano.raponi@opbg.net (M.R.); 2 Post-Graduate School of Occupational Health, Università Cattolica del Sacro Cuore, 00168 Roma, Italy; reparatarosa.diprinzio01@icatt.it (R.R.D.P.); nicola.magnavita@unicatt.it (N.M.); 3 Department of Woman, Child & Public Health, A. Gemelli Policlinic Foundation IRCCS, 00168 Roma, Italy; 4 Italian Ministry of Health, 00153 Roma, Italy; francesco.gilardi13@gmail.com; 5 Health Directorate, Occupational Medicine Unit, Bambino Gesù Children’s Hospital IRCCS, 00146 Roma, Italy; federica.defalco@opbg.net (F.D.F.); mariarosaria.vinci@opbg.net (M.R.V.); vincenzo.camisa@opbg.net (V.C.); annapaola.santoro@opbg.net (A.S.); daniela.casasanta@opbg.net (D.C.); 6 Department of Psychology, European University of Rome, 00163 Roma, Italy; gabriele.giorgi@unier.it

**Keywords:** work-related stress, workplace health promotion, well-being, sickness absence, quality of life, distress, return on investment

## Abstract

Work-related stress is a significant risk for healthcare workers (HCWs). This study aims at evaluating the effectiveness of an individual psychological support programme for hospital workers. In all, 35 workers participated (*n*). A control group of 245 workers (7*n*) was set. Occupational distress was measured by the General Health Questionnaire, (GHQ-12), the quality of life by the Short Form-36 health survey, (SF-36), and sickness absence was recorded. Costs and benefits of the service were evaluated and the return on investment (ROI) was calculated. The level of distress was significantly reduced in the treated group at the end of the follow-up (*p* < 0.001). Quality of life had significantly improved (*p* < 0.003). A 60% reduction of sickness absence days (SADs) following the intervention was recorded. After the treatment, absenteeism in cases was significantly lower than in controls (*p* < 0.02). The individual improvement of mental health and quality of life was significantly correlated with the number of meetings with the psychologist (*p* < 0.01 and *p* < 0.03, respectively). The recovery of direct costs due to reduced sick leave absence was significantly higher than the costs of the programme; ROI was 2.73. The results must be examined with caution, given the very limited number of workers treated; this first study, however, encouraged us to continue the experience.

## 1. Introduction

In post-industrialized countries, traditional occupational diseases are decreasing, whereas nonspecific and multifactorial stress disorders are growing [1]. In European countries a share of 50–60% of all lost working days should be attributed to work-related stress (WRS) [2]. Nonetheless, only a minority of countries in the world include WRS among the risks to be prevented in the workplace [3]. Only about half of the employers inform their workers about psychosocial risks and their effects on health and safety, and less than a third of companies put in place procedures to deal with WRS [4].

Healthcare workers (HCWs) usually face high levels of WRS [5] as a persistent background associated with accident-related spikes [6], which may hinder the quality of the provided healthcare as well as the patient safety [7,8,9]. Epidemiological studies have demonstrated that a high level of WRS is associated with an increased risk of cardiovascular and musculoskeletal diseases, as well as mental disturbances (anxiety, depression, and burnout) [10,11,12,13,14,15,16,17,18]. Many different well-being-promoting interventions for HCWs have been proposed [4,5,9,19]. Organization-directed psychological support is a sharable approach that has already shown its positive effects [7,20]. Individual interventions based on cognitive–behavioural therapy (CBT) have been shown to be effective at some extent [21]. These programmes aim at improving coping strategies, resilience, and control of emotions through various techniques (i.e., individual support, relaxation techniques, focused breath, meditation methods, and self-awareness training) [22,23].

Effectiveness evaluations of Workplace Health Promotion (WHP) programmes, especially psychological support interventions, are not so frequent. One of the most frequently measured objective indicators of HCWs’ health and well-being is sickness absence [5]. Previous evidence showed that, contrary to physical-activity-focused WHP, psychological WHP methods were not considerably related to sickness absenteeism reduction [24]. Subsequently, another study demonstrated that anxiety and depression influence the impact of a perceived health-promotive workplace culture on employee presenteeism and, therefore, productivity [25]. However, an evaluation of the WHP’s economic impact is rarely performed [26]. The importance of WHP economic assessment has also been acknowledged in a very recent systematic review, to the extent that knowing the cost–benefit of WHP interventions helps in defining better political and business solutions for healthier and safer workplaces [27]. In sight of this, the present study aims at evaluating the impact of psychological individual support on HCWs health. In particular, we intended to investigate the specific factors influencing the final result of the WHP in terms of psychological distress and quality of life.

## 2. Materials and Methods

### 2.1. The Help Point (HP) Programme

A WHP plan was developed in our hospital, including a “Help Point” (HP) programme, specifically designed to give psychological support for all employed healthcare personnel who need it. The HP programme was implemented as a part of improvement actions resulting from the assessment of WRS risk and is currently working.

The programme was addressed to all dependent HCWs of the hospital. Participation in the programme was voluntary. HP aimed at preventing work discomfort and, through active listening, making the worker able to analyse the working context, freely express thoughts to colleagues and superiors, and face stressful situations.

The HP path is currently in operation and is led by a multidisciplinary team, which comprises four occupational physicians, one psychologist, and one physician of the Health Directorate, and consists of the six following phases:

1. The demand analysis phase, in which the request of the worker is collected by their occupational physician and then analysed to ascertain the motivational drive (reasons that induced them to ask for HP) through a first meeting led by the occupational physician who has been following the subject during their working life in the hospital and specifically knows their professional context, together with the psychologist who provides an exploratory interview in order to avoid exploitation of the HP programme to achieve particular benefits; to this respect, exclusion criteria are represented by unfitness of psychosocial risk and non-existence of the psychological disorder.
2. The case assessment phase (pre-evaluation), in which the accepted case is carefully assessed to discover the underlying processes of the reported problems and complaints thanks to a combined examination of the psychologist and occupational physicians (through a psychodiagnostic interview and dedicated inspections of the operative unit, respectively).
3. The psychological support phase, in which a series of therapeutic interviews are set up by the psychologist in the form of individual meetings; the duration may vary with respect to both individual status of health and working context and is gradually estimated by the psychologist.
4. The feedback phase (post-evaluation), which comprehends a final interview in which the team analyses whether the achieved outcomes are sufficient to give up the psychological support.
5. The pre-post comparison phase, which allows to compare the initial status to the final status and identify the individual trend on the basis of the collected data; this phase is carried out during the last meeting, after the previous phase.
6. The monitoring phase, in which the subject is being monitored independently by the psychologist and the physicians, using a double-check strategy to evaluate the persistence of improvements.

All team members participate in all phases but the third phase, which is carried out by the psychologist alone. The occupational physicians share an in-depth clinical knowledge of workers and their professional risks, while the Health Directorate physician provides a detailed contextualization in the hospital framework. The coordinating occupational physician supervises the whole process. The final health outcome of the HP path is the improvement of mental health and, thus, of quality of life by gaining functional coping strategies.

### 2.2. Study Design and Setting

In the study period, 35 individuals joined the HP programme. They were mainly women (*n* = 31; 89%), and the mean age was 49.1 years (SD = 8.19), with an average seniority of 22.8 years (SD = 11.89). The prevalent job category was nurse (*n* = 24; 69%) followed by health technician (*n* = 7; 20%); other profiles were physician, biologist, dietician, and social health worker (overall *n* = 4; 11%). Married persons constituted 48.6% of the group, followed by single (22.8%) separated (20%) and divorced (8.6%). Overall 62.9% had children (mean = 1.3). On the whole, 20% performed night work shift and lived out of Rome. The most commonly underlying causes for joining the HP programme were acute distress syndrome (*n* = 25; 71.4%) and, to a lesser, extent work discomfort (*n* = 2; 5.7%).

The analysis was performed from September 2016 to June 2019 in the Bambino Gesù Paediatric Hospital in Rome.

For each of the subjects who asked to participate in the activities of the HP, the following were measured:

1. sociodemographic and work-related variables (age, gender, job category, seniority);
2. clinical management variables related to the performed psychological path (duration of the path, number of meetings);
3. clinical variables (perception of general and mental health level at the beginning and at the end of the treatment), using two questionnaires;
4. sickness absence days (SADs) and sickness absence rate (SAR) in the 6-month and 1-year period before and after the HP programme. The SAR was computed as the ratio between SADs and workable days related to the period (assuming 304 mean workable days in a year and 152 workable days in 6 months);

A control group (7*n* = 245) was also proportionally set through a random selection of dependent workers of the hospital. The inclusion criteria for controls were represented by the following: (1) being a dependent worker of the hospital; and (2) having the same age, gender, and job category of cases. Each case was uniquely matched to seven controls. For the control group, 89% were women and the mean age was 49.1 years (SD = 8.19). They were mainly nurses (7*n* = 168; 69%) and health technicians (7*n* = 49; 20%); physicians, biologists, dieticians, and social health workers were included too (7*n* = 7 for each category; overall, 11%). Married persons constituted 69.8%, followed by single (20.9%) and divorced (9.3%). Overall, 79.1% had children (mean = 2.1). On the whole, 27.8% performed night work shift, and 11.6% lived out of Rome. The mean seniority was 19.25 years (SD = 10.77). They did not have any psychological follow-up nor were they included in other WHP interventions.

### 2.3. The Questionnaires

The questionnaires were self-administered during the evaluation meetings at the beginning and at the end of the HP programme (i.e., the second and the fourth phases listed above).

#### 2.3.1. GHQ-12

The Goldberg’s General Health Questionnaire (GHQ-12) is a 12-item self-administered screening tool used to detect minor psychiatric disorders for the general population [28]. GHQ-12 assesses the current mental state and asks whether that differs from the usual state. The questionnaire focuses on both lack of ability to carry out normal functions and appearance of new distressing phenomena. Each question is ranged on a four-point Likert scale and refers to the last two-week period. The total score can range from 0 to 36 points. Higher scores indicate greater impairment (Cronbach’s alpha for Italian workers: 0.85 [29]); we assumed scores over 21 as needing intervention.

#### 2.3.2. SF-36 Questionnaire

The Short Form-36 Health Survey (SF-36) is a 36-item self-completed investigation of general health [30,31]. Each question is ranged on a five-point Likert scale. SF-36 investigates physical health, general health perception, and psychological–emotional health, and contains eight subscales (domains), each scored from 0 to 100 as a weighted sum of the correspondent questions; two indices are computed deriving from the subscales, synthesizing the overall physical and mental health. The higher the score, the better the perceived level of health (Cronbach’s alpha: 0.88 [32]).

### 2.4. Cost Analysis

SAD-related direct costs were computed using the average per capita cost of a HCW working day (EUR 169.80), which was provided by the Human Resources Directorate of the hospital. The enhancement of savings on total absenteeism in 1 year was used for the definition of the return on investment (ROI) [33], computed as the ratio of the net profit and the investment cost for the HP programme management. In this respect, the weighted sum of the average hourly cost of each working group member was used, multiplied by the number of hours each professional has devoted to the specific activity.

### 2.5. Statistical Analysis

A descriptive analysis was carried out to define the characteristics of the treated population. Pre-treatment variables were compared to post-treatment variables using Student’s paired *t*-test for normally distributed variables or Wilcoxon U test for non-normally distributed variables. Chi-square test was performed between qualitative variables. Two-tailed *p*-value < 0.05 was considered statistically significant.

In order to understand which, among the numerous factors that can influence the outcome of a psychological support intervention, is more important in determining the improvement, we have built two multiple linear regression models using as a dependent variable the pre–post difference in GHQ-12 and, respectively, SF-36 scores and age, gender, job category, seniority, and number of meetings as predictors.

The improvement in GHQ-12 and SF-36 scores was divided at the median. By logistic regression, the association between demographic/social variables and score improvement higher/lower than the median was studied.

Finally, Student’s paired t-test was used to compare SAR between cases and controls. Data were analysed using IBM Statistics Package for Social Sciences (SPSS) (version 25.0).

### 2.6. Ethical Aspects

Our study follows the principles of the Declaration of Helsinki. According to the guidelines on Italian observational retrospective studies, an independent Ethics Committee (EC) approved the study (protocol number 2000/2019). Moreover, as established by the Italian legislation about the obligatory occupational surveillance and privacy management, confidentiality was safeguarded, and informed consent was obtained from all the participants.

## 3. Results

The HP programme lasted approximately 4 months per worker (median = 129 days, IQR = 106–154), distributed on average over eight meetings (median = 8, IQR = 6–13).

Participants in HP programme showed an improvement in the measured parameters. Both GHQ-12 and SF-36 post-test scores significantly improved compared to the pre-test scores (Table 1). All mean scores of the eight subscales of the SF-36 test were higher in post-test compared to the pre-test means. The highest increase was observed in subscale 7 (Emotive Role Limitations: +134.0%), whereas the lowest improvement was in subscale 1 (Physical Activity: +11.7%).

A stepwise multiple linear regression was performed to predict the improvement of mental and general health based on age, gender, job category, seniority, and number of meetings. The pre–post difference of GHQ-12 and of SF-36 scores was used as a dependent variable. The results of the regression indicated that seniority and number of meetings explained 61.5% of the variance of GHQ-12 difference (*p* < 0.003), while the 39.2% of the variance of SF-36 change was significantly predicted by the number of meetings (*p* < 0.03) (Table 2). A significant improvement of mental health was recorded after at least eight meetings (*p* = 0.005).

After the intervention, SADs decreased by 19 days per worker on average in the 1-year period (−60.89%, *p* < 0.04), with a reduction rate (SAR) of 6.43. Absenteeism reduction in the 6-month period was not significantly different among cases. However, the comparison of SAR of the cases with the correspondent controls showed a statistically significant difference (*p* < 0.02) (Table 3).

Moreover, from logistic regression analysis, it emerged that the improvement of quality of life (by SF-36 score) is significantly predicted by the comparison of SAD in the 1-year period (*p* = 0.05).

Regarding the cost analysis, the total amount of hours the working group devoted to HP-related activities was 647 h on average in a year (377 h for the psychologist and 54 h for each physician), which multiplied by the hourly cost of each professional, accounted for EUR 21,556.07 (total cost of investment). The total estimated cost saving related to absenteeism reduction in a year was EUR 80,485.20 (gross profit), the net profit was EUR 58,919.13 (calculated as the difference between the gross profit and the total cost of investment). The ROI was EUR 2.73 for each euro invested.

## 4. Discussion

Workers who voluntarily participated in the HP activities reported a significant reduction in work discomfort and an improvement of mental health status, with an associated reduction of absenteeism. In the treated subjects, SADs decreased by more than 60%, reaching levels lower than the general hospital absenteeism rate. Moreover, the quality of life significantly improved in treated workers. This effect was proportional to the number of meetings, which leads us to believe that the improvement was due to the psychological support interventions. In this respect, eight meetings were enough to realize a noteworthy enhancement of mental health. Additionally, seniority was found to be a predictor of the improvement of mental health. The improvement in productivity generated over EUR 80,000 of savings for the hospital, yielding an ROI of 2.73 for the service. These findings support the effectiveness of the HP activities.

The study confirms the results of the research on this topic. As a part of a comprehensive stress prevention programme existing in the hospital, the psychological support focuses on the individual ability of distress management. The HP programme runs in parallel with an organization-oriented stress intervention based on environmental, ergonomic, structural, and technological improvement measures [34]. Benefits of the HP programme include an outcome relevant for the individual (enhanced psychological and general health) as well as for the organization (reduced sickness absenteeism) [35].

On a clinical level, providing psychological care to employees generates a higher perceived workplace health support, which in turn positively influences productivity [36]. An educational and behavioural mixed approach is confirmed to positively influence cognitive-focused outcomes (such as job-related perceptions) [37]. Moreover, given the structure of the HP path, our study confirms the efficacy of individual engagement of the worker from the beginning of the path, throughout HP development and implementation [38]. In our studied population, age represents the most affecting factor of mental health improvement scores, as older age is associated with a higher risk of stress and emotional exhaustion [39], even if common mental disorders are an age-independent global disease burden [40]. Moreover, the prevalence of women is particularly higher in the studied population than in the whole hospital population (90.3% vs. 71.0%). Women are more susceptible to stress-related disorders than men and have a different neural processing of control [41]. Indeed, women are more prone to seek psychological support from primary care services (such as the occupational service), especially in the case of WRS [42,44]. Considering that different categories of psychiatric diseases are differently distributed among women and men [42], this composition bias could alter the picture of the mental status of workers in the hospital. Participation of male workers should be encouraged, for instance, by adding a fast-screening questionnaire for the most common male mental disorders during the sanitary surveillance. Finally, current evidence does not definitely assess the duration of CBT to be effective [21]. Our results suggest at least eight meetings on average for a considerably improved mental health condition.

At the organization level, sickness absenteeism is a reliable indicator of WRS and quality of life [5]. Our results proved that the HP programme notably lessened absenteeism, and this effect could last up to a year after the end of the programme. This finding supports sickness absence days to be a reliable objective indicator also in a medium-term period. As established in a previously evaluated WHP addressed to the same population [34,43], improved general and mental health leads to recovered working days, a remarkable economic saving for the hospital.

Furthermore, this study sharpens the specific role of two professional figures. On the one hand, the occupational physician, who embodies the figure of a professional mediator of the needs of workers and the organization and is in the meantime the solely responsible for the health of workers by the Italian law [44]. On the other hand, the psychologist, who is called to take care of the mental aspect of the workforce. Both these professionals need to raise the awareness of workers on the multilayered beneficial effects of the programme and the importance of steadiness in following the path throughout its duration as an individual empowerment strategy able to solve the underlying criticality.

To the best of our knowledge, this is the first effectiveness study of a hospital psychological support service conducted in Italy. The main strength is the accomplished effectiveness of a relatively short psychological support intervention on a medium-term period at the clinical and organization levels. The main limitation of the study is the small sample. The results need further confirmation through a larger number of observations possibly in a longer follow-up.

## 5. Conclusions

The psychological support programme showed a consistent effectiveness on mental health and quality of life as well as on productivity related to a decreased absenteeism in healthcare settings. It may be considered an effective and cost-saving approach able to mitigate the risk of WRS, a risk that, however, cannot be completely eradicated. The main future challenge in this field lies in confirming the validity of the preliminary results highlighted here on a wide-ranging workforce also in non-healthcare settings.

## Figures and Tables

**Table 1:** GHQ-12 and SF-36 mean scores before and after treatment.

Variable	Pre-HP Score (Mean ± SD)	Post-HP Score (Mean ± SD)	Difference (Mean ± SD)	Paired *t* Test for Groups (*p* Value)
GHQ-12	20.90 ± 7.88	7.23 ± 4.82	−13.67 ± 7.88	0.000
SF-36	375.69 ± 156.50	575.14 ± 140.97	199.45 ± 131.09	0.000
Subscale 1—Physical Activity	77.10 ± 24.61	86.13 ± 16.73	9.43 ± 21.74	0.015
Subscale 2—Physical Role Limitations	47.81 ± 36.46	82.90 ± 22.14	31.94 ± 31.35	0.000
Subscale 3—Physical Pain	52.03 ± 28.56	73.13 ± 23.89	21.09 ± 23.87	0.000
Subscale 4—General Health	55.71 ± 21.71	69.97 ± 19.49	13.51 ± 20.14	0.000
Subscale 5—Vitality	35.16 ± 17.93	60.68 ± 20.12	26.03 ± 19.91	0.000
Subscale 6—Social Activities	39.19 ± 20.55	70.16 ± 22.65	30.23 ± 25.42	0.000
Subscale 7—Emotive Role Limitations	34.86 ± 34.44	81.58 ± 27.57	12.31 ± 27.44	0.000
Subscale 8—Mental Health	39.74 ± 15.70	69.94 ± 16.29	29.20 ± 17.87	0.000

**Table 2:** Stepwise linear regression analysis. Relationship between demographic and intervention-related variables and improvement.

Variable	GHQ-12		SF-36	
	Standardized Beta	*p* Value	Standardized Beta	*p* Value
Seniority	0.548	0.001	-	-
Number of meetings	−0.483	0.002	0.392	0.022
Determination coefficient of the model (R^2^)	0.379	0.000	0.154	0.022

Variables excluded from the model: gender, age, job category.

**Table 3:** Changes in sickness absenteeism rate.

		Pre, 6 Months (Mean ± SD)	Post, 6 Months (Mean ± SD)	*p*	Pre, 12 Months (Mean ± SD)	Post, 12 Months (Mean ± SD)	*p*
Cases	SADs	20 ± 49	8 ± 16	0.144	32 ± 50	13 ± 20	0.039
	SAR	6.62	2.68		10.56	4.23	
Controls	SADs	6 ± 13	3 ± 7	0.002	13 ± 23	7 ± 13	0.001
	SAR	1.98 ± 4.40	1.02 ± 2.27		4.17 ± 7.54	2.29 ± 4.13	
Cases vs. controls (reduction)	SAD	−(12 ± 47) vs. −(3 ± 14)		0.019	−(19 ± 49) vs. −(6 ± 24)		0.016
	SAR	−(3.94 ± 15.34) vs. −(0.95 ± 4.64)			−(6.34 ± 16.07) vs. −(1.88 ± 8.06)		

## Data Availability

The data presented in this study are available on request from the corresponding author. The data are not publicly available due to ethical restrictions.
